# A Delicate Matter: The Burden of Frailty on AMI-CS∗

**DOI:** 10.1016/j.jacadv.2024.100947

**Published:** 2024-05-15

**Authors:** J. Antonio Gutierrez, Dennis I. Narcisse, Jonathan N. Menachem

**Affiliations:** a Division of Cardiology, Duke University Medical Center, Durham, North Carolina, USA; b Duke Clinical Research Institute, Durham, North Carolina, USA; c Durham VA Medical Center, Durham, North Carolina, USA; d Division of Cardiology, Vanderbilt University Medical Center, Nashville, Tennessee, USA

**Keywords:** acute myocardial infarction, elderly, frailty, heart failure

**Figure:**
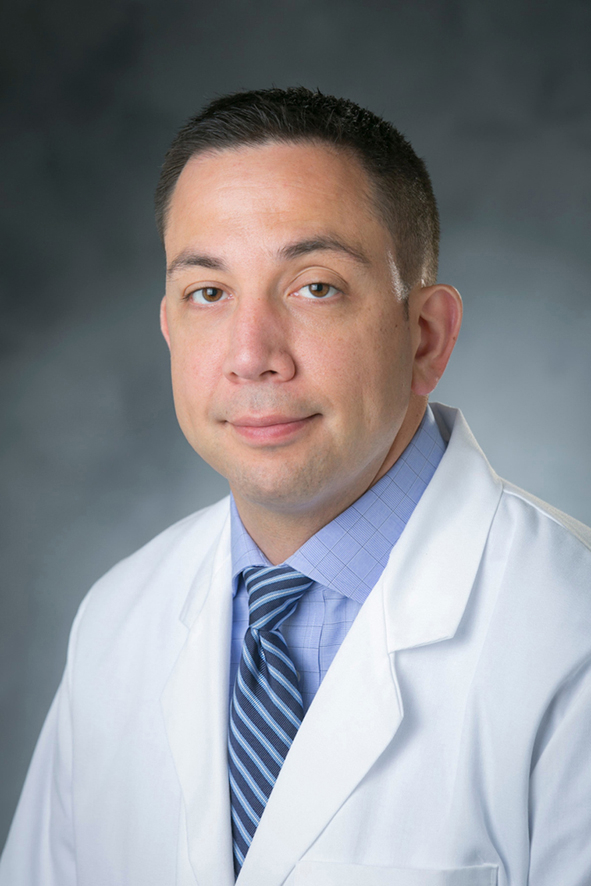

Frailty, a term frequently encountered in the realm of geriatric medicine, has become a critical determinant in the prognosis of patients with cardiovascular diseases, including acute myocardial infarction (AMI) and heart failure (HF). This concept, while simple in its essence, is a complex interplay of decreased physiological reserve and increased vulnerability to adverse health outcomes across multiple organ systems. The frailty piece of the geriatric syndrome, often characterized by a constellation of physical, cognitive, and functional impairments, is a key marker of vulnerability that significantly influences not only outcomes but also clinical decision-making. The definitions of frailty, though varied, commonly converge on the presence of reduced strength, endurance, and physiological function. Frailty is assessed through several validated scales and indices, each aiming to quantify the severity and its potential impact on health outcomes. Among these, the Frailty Index, Hospital Frailty Risk Score, and Physical Frailty Phenotype are widely recognized for their predictive value in clinical settings.^1^, ^2^, ^3^ These assessment tools have been validated in prior studies examining the influence frailty exerts on the outcomes of cardiovascular disease. The negative impact of frailty on outcomes could be related to medication intolerance, procedural complications, and notably, a heightened risk of in-hospital mortality.^4^

As the global demographic shifts toward an aging population, the prevalence of frailty and its implications in the context of acute cardiovascular events have become prominent. Older adults, who represent a growing proportion of patients presenting with AMI and HF, are particularly susceptible to the deleterious effects of frailty. Prior work has shown that adherence to guideline recommendations for the management of AMI in both older people and those with multiple comorbidities lower compared to younger patients. This is coupled with evidence showing that people with frailty are less likely to receive invasive coronary angiography or revascularization and had longer hospitalizations, contributing to poorer outcomes.^5^ The impact of frailty has also been examined in HF studies, such as the prospective registry FRAGILE-HF (Prevalence and Prognostic Value of Physical and Social Frailty in Geriatric Patients Hospitalized for HF), found there were multiple frailty domains prevalent in hospitalized elderly patients with HF which negatively impacted outcomes.^6^^,^^7^ These effects were even more exaggerated in frail patients presenting decompensated HF.

AMI complicated by cardiogenic shock (AMI-CS) is the most severe presentation of AMI and yields high morbidity and mortality. Despite significant advances in therapeutic and supportive measures, AMI-CS continues to be characterized by dramatically high mortality rates, upward of 40%.^8^^,^^9^ This statistic is influenced by several factors, including patient-specific baseline characteristics, shock severity, the extent of organ dysfunction, and the response to therapeutic interventions. Frailty, within this context, emerges as a potential amplifier of risk, warranting closer examination of its role in the outcomes of AMI-CS. While age has traditionally been a surrogate for frailty, the complexity of geriatric syndromes underscores the need to explore frailty as an independent predictor. Prior studies have shown that older adults, despite their advanced age, can benefit from coronary revascularization, suggesting that age alone should not preclude aggressive therapeutic interventions.^10^^,^^11^ However, the intricate relationship between frailty, geriatric syndromes, and cardiovascular interventions complicates the clinical approach to managing AMI-CS in frail patients. On top of this, the challenge of studying frailty in the context of AMI-CS is compounded by the exclusion of patients with geriatric conditions from observational studies and registries. This has created a knowledge gap in understanding the full impact of frailty on AMI-CS.^12^

In the paper by Jamil et al^13^ in this issue of *JACC: Advances*, the authors should be congratulated on taking on an understudied cohort to bring forth significant insights into the compounded vulnerability of frail patients with AMI-CS. A substantial portion of AMI-CS hospitalizations involves frail individuals, who are subsequently faced with significantly higher in-hospital mortality rates. This correlation not only highlights frailty as a critical marker of vulnerability but also suggests its contributory role in worsening outcomes for AMI-CS. This finding warrants better understanding of frailty’s impact on AMI-CS trajectory and the mechanisms through which it exacerbates the shock state in this vulnerable patient population. In addition, the study draws attention to the trend of frail patients being less likely to receive aggressive interventions, such as coronary revascularization or mechanical circulatory support. This observation raises critical questions about the clinical decision-making processes and where there is possible reluctance or conservatism in offering invasive treatments to frail patients. The underlying reason for this trend calls for further exploration to ensure that treatment decisions are not improperly influenced by frailty status but are instead guided by a balanced consideration of risks and benefits.

However, this study is not without limitations which are important to highlight. A notable gap is the absence of data on posthospitalization outcomes, particularly regarding the quality of life of the frail patients surviving to hospital discharge. This lack of data presents a significant challenge in fully comprehending the short-term aftermath of AMI-CS treatment in frail individuals. Understanding the recovery or potential decline following hospital discharge is paramount for crafting comprehensive care strategies that extend beyond the acute hospitalization phase. Additionally, it is unclear of the reliability of frailty assessment in the setting of AMI-CS which poses a significant challenge. Given that many patients presenting with AMI-CS are frail at baseline and further exacerbated in the acute setting, the timing of measurement and accuracy are unclear. Future work in this area should focus on identifying a uniform assessment criterion to best understand frailty within the AMI-CS population.

The profound complexity of frailty intersecting with AMI-CS highlights a need for personalized management strategies in shock care. Recognizing the distinct outcomes between frail and nonfrail patients, future work should focus on tailoring treatment approaches to ensure that frail patients receive interventions that are both safe and beneficial. Furthermore, incorporating comprehensive geriatric assessments into the care framework of shock teams could be a strategy implemented in this patient population. Given the prevalence, the expertise of geriatricians or geriatric cardiology specialists could be invaluable and provide opportunity for in-hospital and longitudinal care. These providers should be skilled in assessing the broader implications of geriatric syndromes, including frailty, and their insights could guide more informed, holistic treatment decisions. Future work should explore the impact of integrating geriatric assessments on patient outcomes, potentially setting new standards for managing frail patients with AMI-CS to provide opportunity to standardize care.

In conclusion, frailty and AMI-CS represent a profound challenge for cardiologists given the markedly elevated mortality rates despite contemporary management of coronary disease and shock. The insights gained from this study highlight some opportunity for future work to potentially improve clinical practice. Tailored management strategies are essential to create care pathways for this vulnerable patient population. Additionally, the integration of comprehensive geriatric assessments into the treatment of CS may offer clinicians deeper understanding to patient status. By incorporating a multidisciplinary approach that includes geriatric expertise, clinicians will be better equipped to provide the most beneficial treatment options. Future work must address the current gaps in knowledge, which include frailty assessments in shock and postdischarge outcomes, to improve care in our aging and/or frail population who present with AMI-CS.

## Funding support and author disclosures

Dr Gutierrez has received research funding from the Veterans Health Administration. All other authors have reported that they have no relationships relevant to the contents of this paper to disclose.
